# The relationship between contrast-enhanced computed tomography radiomics features and mitosis karyorrhexis index in neuroblastoma

**DOI:** 10.1007/s12672-024-01067-0

**Published:** 2024-06-01

**Authors:** Xin Chen, Haoru Wang, Yuwei Xia, Feng Shi, Ling He, Enmei Liu

**Affiliations:** 1 Department of Radiology, Children’s Hospital of Chongqing Medical University, National Clinical Research Center for Child Health and Disorders, Ministry of Education Key Laboratory of Child Development and Disorders, Chongqing Key Laboratory of Child Neurodevelopment and Cognitive Disorders, Chongqing, 400014 China; 2 Shanghai United Imaging Intelligence, Co., Ltd, Shanghai, 200030 China; 3 Department of Respiratory Medicine, Children’s Hospital of Chongqing Medical University, National Clinical Research Center for Child Health and Disorders, Ministry of Education Key Laboratory of Child Development and Disorders, Chongqing Key Laboratory of Child Rare Diseases in Infection and Immunity, Chongqing, 400014 China

**Keywords:** Neuroblastoma, Computed tomography, Radiomics, Mitosis karyorrhexis index

## Abstract

**Objective:**

Mitosis karyorrhexis index (MKI) can reflect the proliferation status of neuroblastoma cells. This study aimed to investigate the contrast-enhanced computed tomography (CECT) radiomics features associated with the MKI status in neuroblastoma.

**Materials and methods:**

246 neuroblastoma patients were retrospectively included and divided into three groups: low-MKI, intermediate-MKI, and high-MKI. They were randomly stratified into a training set and a testing set at a ratio of 8:2. Tumor regions of interest were delineated on arterial-phase CECT images, and radiomics features were extracted. After reducing the dimensionality of the radiomics features, a random forest algorithm was employed to establish a three-class classification model to predict MKI status.

**Results:**

The classification model consisted of 5 radiomics features. The mean area under the curve (AUC) of the classification model was 0.916 (95% confidence interval (CI) 0.913–0.921) in the training set and 0.858 (95% CI 0.841–0.864) in the testing set. Specifically, the classification model achieved AUCs of 0.928 (95% CI 0.927–0.934), 0.915 (95% CI 0.912–0.919), and 0.901 (95% CI 0.900–0.909) for predicting low-MKI, intermediate-MKI, and high-MKI, respectively, in the training set. In the testing set, the classification model achieved AUCs of 0.873 (95% CI 0.859–0.882), 0.860 (95% CI 0.852–0.872), and 0.820 (95% CI 0.813–0.839) for predicting low-MKI, intermediate-MKI, and high-MKI, respectively.

**Conclusions:**

CECT radiomics features were found to be correlated with MKI status and are helpful for reflecting the proliferation status of neuroblastoma cells.

**Supplementary Information:**

The online version contains supplementary material available at 10.1007/s12672-024-01067-0.

## Introduction

Neuroblastoma is a prevalent solid tumor among children and a major cause of cancer-related deaths in this population. This disease is highly heterogeneous, and patient prognosis varies significantly [1, 2]. The International Neuroblastoma Pathology Classification (INPC) categorizes neuroblastoma into risk groups based on patient age, histological subtype, and mitosis-karyorrhexis index (MKI) [3]. MKI refers to the proportion of cells exhibiting mitosis or nuclear fragmentation in a population of 5000 tumor cells. The three classifications based on MKI status are low-MKI, intermediate-MKI, and high-MKI [4, 5]. Therefore, MKI can reflect the proliferation status of neuroblastoma cells. Studies have shown that MKI is an independent predictor of neuroblastoma prognosis, with high-MKI indicating greater malignancy and poorer prognosis [6, 7]. Biopsy is the gold standard for determining MKI. However, it is invasive and has limitations. Accurately counting MKI can be challenging due to sampling errors and the subjectivity of manual counting, and biopsy can also impact the pathological diagnosis of neuroblastoma due to spatial and temporal heterogeneity within the tumor [8–10]. Therefore, investigating the relevant biomarkers associated with MKI status in neuroblastoma is necessary to reflect the proliferation status of neuroblastoma cells.

Radiomics is a high-throughput image analysis technique that involves extracting a large number of features from radiological images to describe tumor characteristics. These features can be then used in machine learning to develop prediction models for deeper analysis and identification of medical images. Radiomics offers a non-invasive method for extracting imaging features that cannot be identified by the naked eye from medical images, providing a convenient way to assess prognosis and predict treatment outcomes [11–13]. This technique has been extensively studied in both adult and pediatric solid tumors and has demonstrated its ability to better reflect the pathological basis and clinical features of lesions, making it a promising imaging biomarker for future clinical applications [14–17]. Radiomics is also expected to be used to determine the temporal and spatial heterogeneity of pediatric neuroblastoma. Previous studies have demonstrated that radiomics can be helpful in revealing the MYCN gene amplification status of neuroblastoma, determining the primary site response to neoadjuvant chemotherapy, identifying high-risk subgroup, and predicting prognosis [18–21]. Feng et al. [22] discovered a significant correlation between radiomics features extracted from ^18^F-FDG PET/CT and MKI status of neuroblastoma, which enables the differentiation between low-MKI and non-low-MKI. Nevertheless, ^18^F-FDG PET/CT is not commonly used as an initial diagnostic tool for neuroblastoma, and their study only divided neuroblastoma into low-MKI and non-low-MKI, making it less effective in distinguishing between high-MKI and intermediate-MKI.

Contrast-enhanced computed tomography (CECT) is commonly used for delineating vascular structures of neuroblastoma before surgery. Compared with venous-phase CECT images, arterial-phase CECT images may better reflect the heterogeneity of neuroblastoma lesions. Therefore, the objective of this study was to investigate the arterial-phase CECT radiomics features associated with the MKI status in neuroblastoma.

## Materials and methods

### Patients

**Fig. 1:**
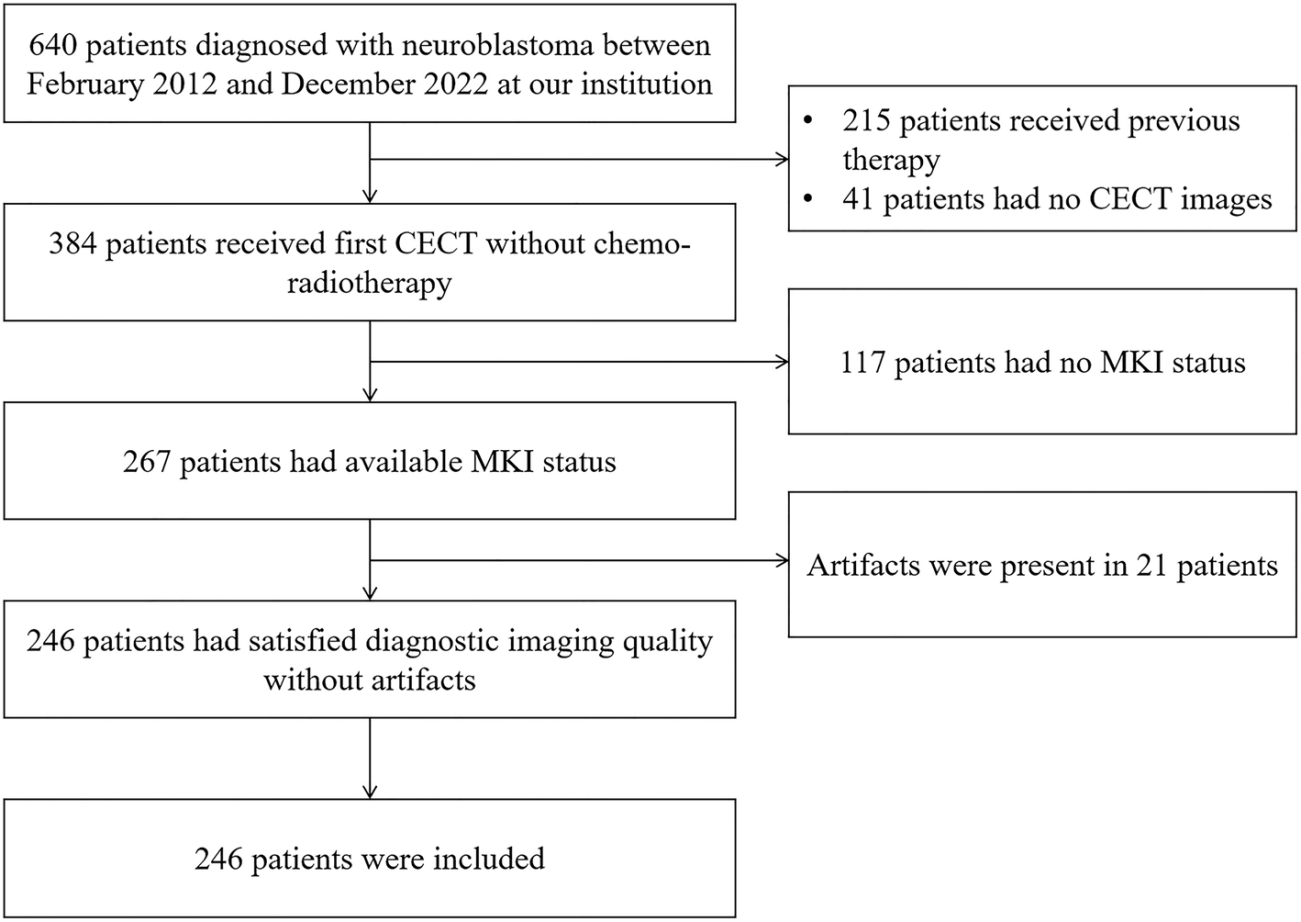
The process of patient selection

CECT imaging data from 246 patients diagnosed with neuroblastoma at our hospital from February 2012 to December 2022 were retrospectively collected. The patients were divided into three groups based on their MKI status (low = 83, intermediate = 80, high = 83) and randomly stratified into a training set and a testing set at a ratio of 8:2. To be eligible for inclusion in this retrospective study, patients had to meet the criteria of confirmed neuroblastoma with MKI status on their pathological report, first CECT without chemo-radiotherapy, and complete CECT imaging data with satisfied diagnostic imaging quality without artifacts. Patients with incomplete CECT imaging data or clinical information, as well as those who had undergone CECT with previous chemo-radiotherapy were excluded. The process of patient selection is detailed in Fig. 1.

This study adhered to the CheckList for EvaluAtion of Radiomics research (CLEAR) endorsed by the European Society of Radiology [23]. The filled checklist is listed in the Supplementary Table 1. The radiomics quality score (QRS) was used to assess the report of this study [24]. This retrospective study was approved by the Institutional Review Board of Children's Hospital of Chongqing Medical University, and a waiver of patient consent to participate was obtained. All methods were performed in accordance with the relevant guidelines and regulations. Based on the count of cells displaying mitosis or nuclear fragmentation within a population of 5,000 tumor cells, MKI status was categorized into three groups: low-MKI (< 100/5000 cells or < 2%), intermediate-MKI (100 to 200/5000 cells or 2% to 4%), or high-MKI (> 200/5000 cells or > 4%) [22].

### Image acquisition

Children were examined in a calm state, and those who could not cooperate were sedated by professional anesthesiologists. The VCT 64 slice spiral CT (GE Healthcare, USA) scanner or the Brilliance iCT spiral CT (Philips, Netherlands) scanner was used to obtain arterial-phase CECT images. Tube voltage: 80–120 kV; tube current: 120–200 mAs; slice thickness: 5.0 mm; slice spacing: 5.0 mm. Nonionic iodinated contrast agent (Omnipaque 300 mg I/mL or Visipaque 320 mg I/ml, GE Healthcare) was used. The procedure involved injecting a contrast agent (1.5 ml–2 ml/kg of body weight) into the patient’s anterior brachial vein at a rate of 1–3 ml/s, followed by performing arterial-phase CECT scan at 15–30 s after the injection, respectively.

### Image segmentation

**Fig. 2:**
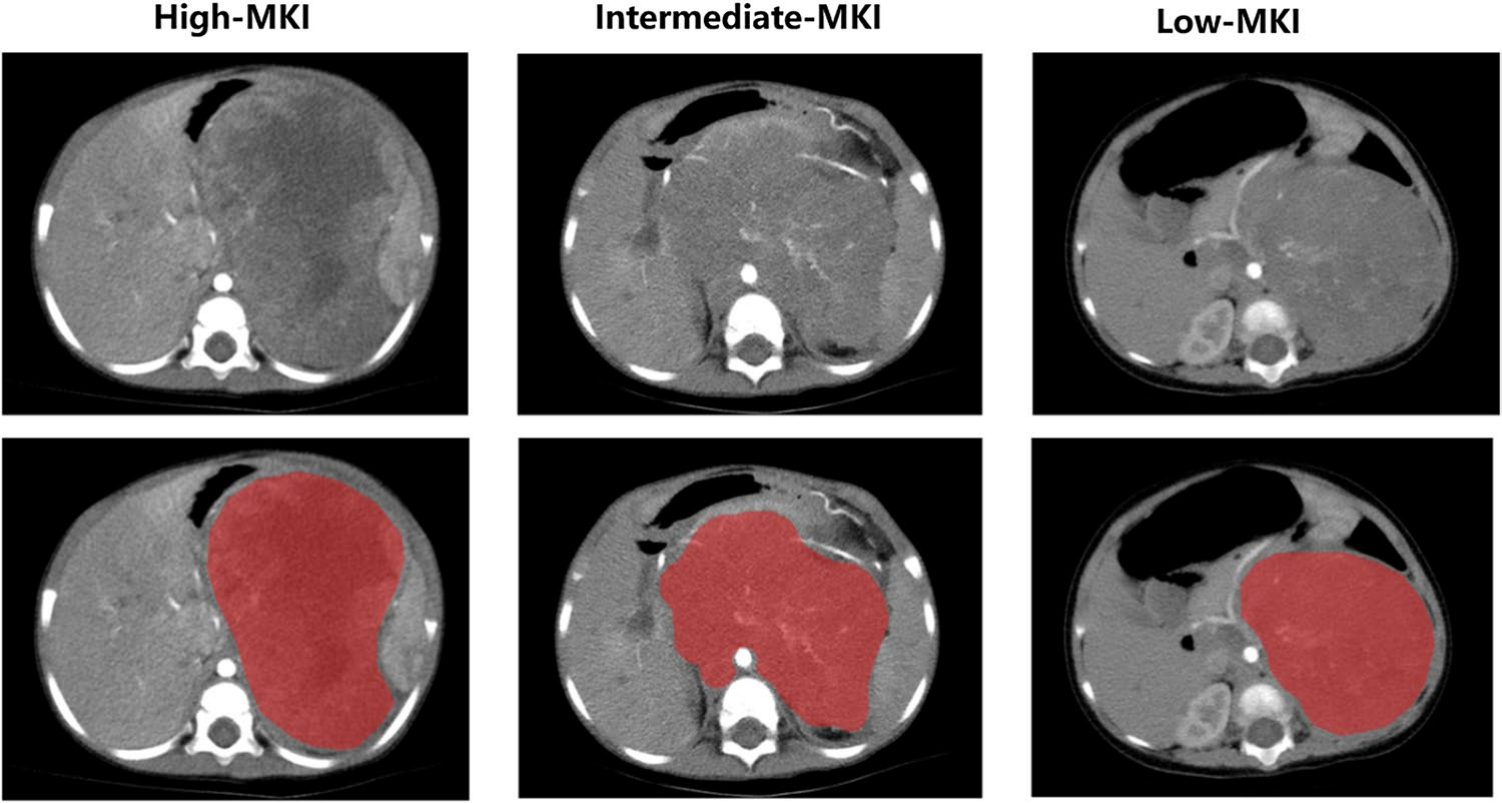
Examples of tumor region of interest delineation

The initial step of the radiomics analysis included uploading the CECT images of all patients to a research platform called uAI Research Portal (version 20240130, https://urp.united-imaging.com/) [25]. In this study, we performed both consensus-based segmentation and reliability analysis [26]. Regarding the consensus-based segmentation, a radiologist with 3 years of experience initially manually delineated the whole tumor region of interest (ROI) on arterial-phase CECT images, layer by layer. Subsequently, a three-dimensional ROI was generated. Another radiologist with 15 years of experience in tumor imaging validated all segmented ROIs (Fig. 2). Regarding the reliability analysis, 30 randomly selected cases included 10 cases from each of the low, intermediate, and high MKI groups from the training set to evaluate the inter-observer agreement of radiomics features, and the ROIs were re-segmented by the radiologists with 5 and 10 years of experience in pediatric imaging. The intra-class correlation coefficient (ICC) for the radiomics features extracted from the ROIs delineated in two separate segmentations was calculated. In this study, an ICC of type (2, 1) was used to assess reproducibility. This model, also known as the two-way random effects model for single measurement with absolute agreement, provides a comprehensive measure of both consistency and accuracy in the ratings [27].

### Radiomics analysis

**Fig. 3:**
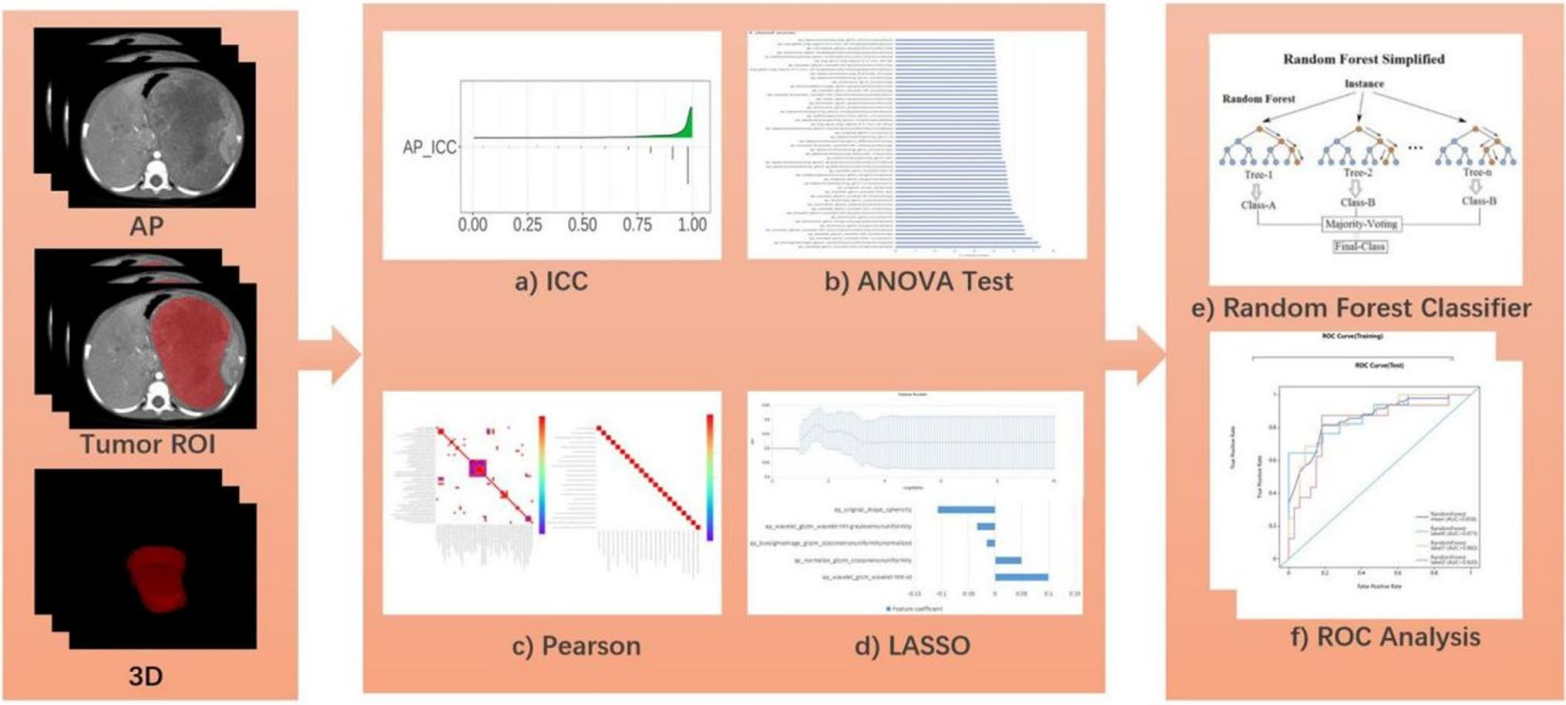
Workflow of radiomics pipeline used in this study

The radiomics analysis pipeline is detailed in Fig. 3. To standardize the voxel sizes, all CECT images were resampled using the B-spline interpolation algorithm to a voxel spacing of 1 mm * 1 mm * 1 mm, and a fixed bin number of 64 was also applied. Utilizing a fixed bin number in the discretization process before radiomics feature extraction aids in reducing the influence of noise while preserving the crucial textures of the regions of interest in the image [28]. Following image preprocessing, a total of 2264 radiomics features were automatically extracted from each ROI. In this study, PyRadiomics (version 3.0.1, https://pyradiomics.readthedocs.io/en/) was embedded in the uAI Research Portal software, so the extracted original radiomics features were all adhered to image biomarker standardization initiative (IBSI) [29].

The first-order statistics group contained 18 features, such as energy, entropy, kurtosis, skewness, and percentiles (e.g., 10th and 90th). The shape-based features group contained 14 features, such as sphericity, surface area, voxel volume, and maximum 3D diameter. The texture features group included 72 features derived from different texture analysis methods, such as 21 gray level co-occurrence matrix (GLCM) features, 16 gray level run length matrix (GLRLM) features, 16 gray level size zone matrix (GLSZM) features, 5 neighboring gray tone difference matrix (NGTDM) features, and 14 gray level dependent matrix (GLDM) features, which can quantify regional heterogeneity differences. The study also applied 24 filters (Box Mean, Additive Gaussian Noise, Binomial Blur, Curvature Flow, Box-sigma, Normalize, Laplacian Sharpening, Discrete Gaussian, Mean, Speckle Noise, Recursive Gaussian, Shot noise, LoG (sigma: 0.5, 1, 1.5, 2), and Wavelet (LLL, LLH, LHL, LHH, HLL, HLH, HHL, HHH)) to obtain derived images, and then extracted first-order statistics and texture features based on the derived images (2160 derived features). The radiomics features were standardized using z-score normalization.

First, the radiomics features with an ICC of greater than 0.80 were considered to have agreeable reproducibility and were selected for further analysis. After assessing feature reproducibility, the study carried out three steps of feature selection in the training set. The features were statistically compared between groups using an ANOVA test, and a *P*-value cutoff of 0.05 was applied to obtain the top 50 significant radiomics features. Then, the radiomics features with a Pearson correlation coefficient greater than 0.90 were discarded to eliminate the highly correlated features. Furthermore, the most relevant radiomics features were selected using the least absolute shrinkage and selection operator (LASSO) method, with the optimal hyperparameter selected through a fivefold cross-validation. Lastly, a final random forest radiomics model was trained based on the retained features, and applied to the testing set. Additionally, a random forest radiomics model was trained to determine the predictive performance of individual radiomics features. The hyperparameters of the random forest model were determined using an automated method of hyperparameter optimization. Specifically, GridSearchCV from the ‘scikit-learn’ package was employed. The parameters of the random forest algorithm were configured as: criterion = entropy, max depth = 64, min samples leaf = 6, min samples split = 7, N estimators = 80, threshold = 0.5. In our study, we approached the three-class classification problem through a 'One versus Rest' strategy. This method essentially breaks down a multiclass problem into multiple binary problems. In this case, for our three-class problem, it led to three binary classification tasks. Each binary problem was tackled by considering one class versus the rest combined.

### Statistical analysis

The statistical analysis was performed using SPSS software (version 26.0) and uAI Research Portal (version 20240130, https://urp.united-imaging.com/) software. Specifically, the Scikit-Learn library, an open-source machine learning library, was utilized to build the random forest model. The versions and dependencies were: Python: 3.7; Scikit-Learn: 0.23.2; Numpy: 1.19.2; Pandas: 1.1.3; Matplotlib: 3.3.2. ANOVA test was used for comparing quantitative data between the three groups, and chi-square test was used for comparing qualitative data between the three groups. A *P*-value less than 0.05 considered statistically significant. The diagnostic performance of the three-class classification radiomics model was evaluated using receiver operating characteristic (ROC) curve analysis. Area under the curve (AUC), 95% confidence interval (CI), accuracy, specificity, and sensitivity of the radiomics model were calculated. The clinical utility analysis of the radiomics model for predicting MKI status in the training and testing sets was made through decision curve analysis (DCA).

## Results

### Patient characteristics

A total of 246 neuroblastoma patients were retrospectively included, and there were 135 males and 111 females, with an age range of 1 month to 13 years (mean age: 2.32 years, standard deviation: 2.35 years). After stratifying the patient cohort, there were 197 cases in the training set and 49 cases in the testing set. Table 1 presents the clinical characteristics of the included patients, and no significant differences were observed in most of patient characteristics among the low-MKI, intermediate-MKI, and high-MKI groups (*P* > 0.05). The overall RQS of this study was 16, and the filled checklist is shown in Supplementary Table 2.

**Table 1:** The clinical characteristics of the included patients

Characteristics	Overall	Low-MKI	Intermediate-MKI	High-MKI	*P* value
Entire cohort (n = 246)					
Age (mean ± SD, years)	2.3 ± 2.3	2.5 ± 2.9	2.1 ± 2.0	2.4 ± 2.0	0.444
Sex					0.317
Female	111	43	34	34	
Male	135	40	46	49	
Calcification	198	61	67	70	0.142
Across midline	177	55	54	68	0.045
VMA (mean ± SD)	17.0 ± 29.4	11.3 ± 19.4	20.6 ± 31.4	19.3 ± 34.9	0.154
Training set (n = 197)					
Age (mean ± SD, years)	2.4 ± 2.5	2.6 ± 3.0	2.2 ± 2.2	2.5 ± 2.1	0.551
Sex					0.129
Female	86	34	29	23	
Male	111	32	35	44	
Calcification	159	48	53	58	0.113
Across midline	141	44	43	54	0.131
VMA (mean ± SD)	16.1 ± 26.0	11.0 ± 19.4	22.5 ± 30.9	15.8 ± 26.6	0.087
Testing set (n = 49)					
Age (mean ± SD, years)	1.9 ± 1.8	1.9 ± 2.3	1.6 ± 1.3	2.1 ± 1.8	0.681
Sex					0.382
Female	25	9	5	11	
Male	24	8	11	5	
Calcification	39	13	14	12	0.929
Across midline	36	11	11	14	0.141
VMA (mean ± SD)	20.4 ± 40.4	12.8 ± 20.6	14.7 ± 33.4	34.5 ± 58.1	0.353

*SD* standard deviation, *VMA* Vanillylmandelic Acid

### Radiomics feature selection

**Fig. 4:**
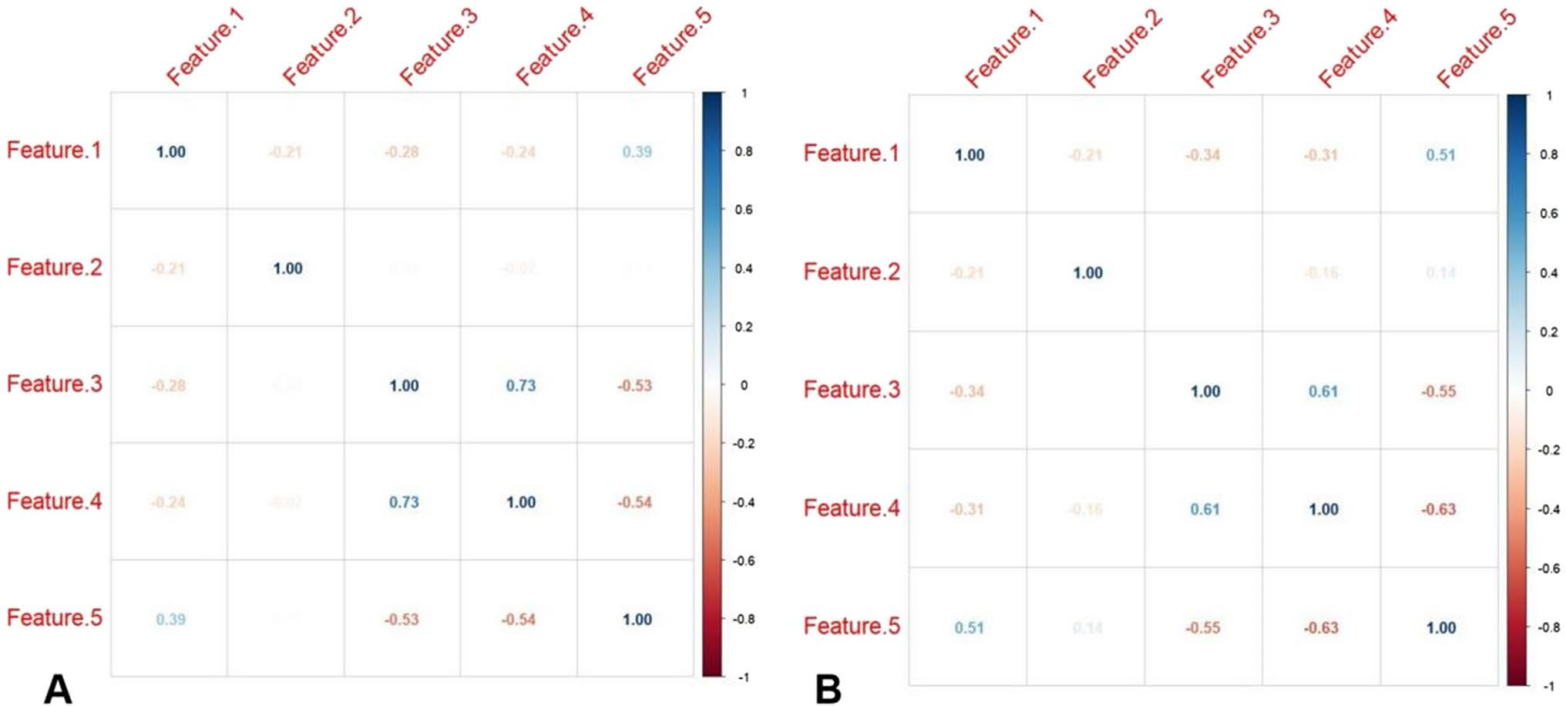
Correlation coefficient map of the final selected radiomics in the training set (**A**) and testing set (**B**). Features 1 to 5 represent boxsigmaimage_glszm_SizeZoneNonUniformityNormalized,wavelet_glcm_wavelet-HHH-Idm,wavelet_glszm_wavelet-HHL-GrayLevelNonUniformity,normalize_glszm_SizeZoneNonUniformity,original_shape_Sphericity, respectively

Initially, 2264 radiomics features were extracted from each ROI. Out of these, 2011 features were deemed to have acceptable inter-observer and intra-observer reproducibility (i.e., ICC > 0.8). Next, 50 radiomics features with a smaller *P*-value were retained through the ANOVA test. Then, 33 radiomics features with a Pearson correlation coefficient greater than 0.90 were removed, resulting in the retention of only 17 features. Finally, in the LASSO algorithm, 5 radiomics features with non-zero coefficients were identified and selected (Fig. 4).

### Predictive performance of individual radiomics features

**Fig. 5:**
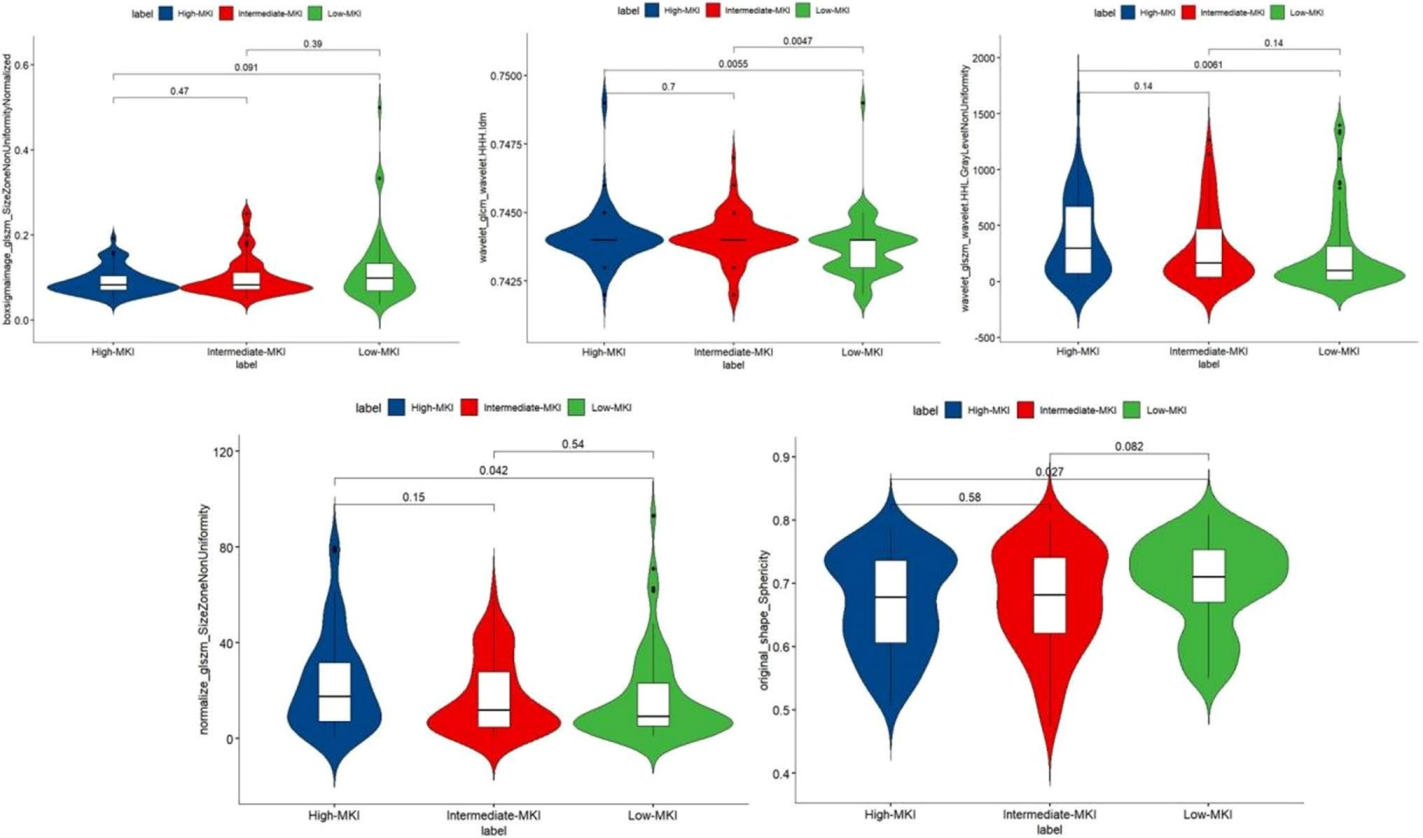
Multiple comparison of the final selected radiomics among different groups in the training set

**Fig. 6:**
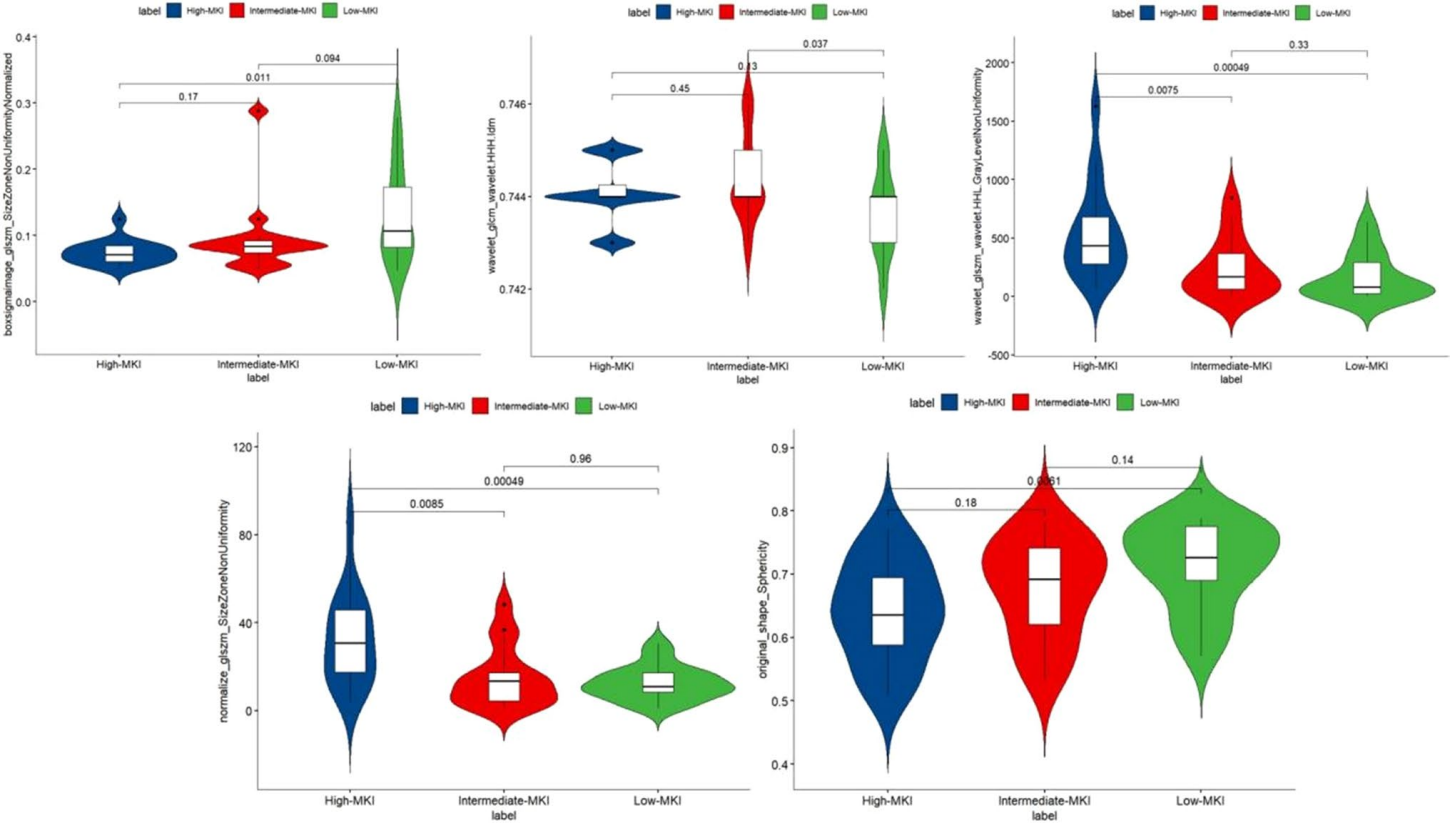
Multiple comparison of the final selected radiomics among different groups in the testing set

Among the final selected radiomics features, wavelet-HHH_GLCM_Idm based radiomics model achieved the highest mean AUC of 0.812 (95% CI 0.738–0.885) in the training set and 0.816 (95% CI 0.656–0.941) in the testing set. Besides, shape_sphericity also had a moderate mean AUC of 0.807 (95% CI 0.731–0.882) in the training set and 0.709 (95% CI 0.643–0.768) in the testing set. Table 2 shows the predictive performance of individual radiomics features. Figures 5 and 6 illustrate the multiple comparison of the final selected radiomics features among the three groups in the training set and testing set, respectively.

**Table 2:** Diagnostic performance of individual radiomics features

Radiomics feature	Filter	Category	Mean AUC_training	Mean AUC_testing
Idm	wavelet-HHH	GLCM, texture	0.812 (95%CI 0.738–0.885)	0.816 (95%CI 0.656–0.941)
Sphericity	original	shape	0.807 (95%CI 0.731–0.882)	0.709 (95%CI 0.643–0.768)
Size zone non uniformity	normalize	GLSZM, texture	0.720 (95%CI 0.644–0.795)	0.653 (95%CI 0.580–0.813)
Gray level non uniformity	wavelet-HHL	GLSZM, texture	0.706 (95%CI 0.630–0.782)	0.609 (95%CI 0.435–0.774)
Size zone non uniformity normalized	boxsigmaimage	GLSZM, texture	0.721 (95%CI: 0.645–0.796)	0.588 (95%CI: 0.415–0.751)

*GLCM* gray level co-occurrence matrix, *GLSZM* gray level size zone matrix

### Model performance

**Fig. 7:**
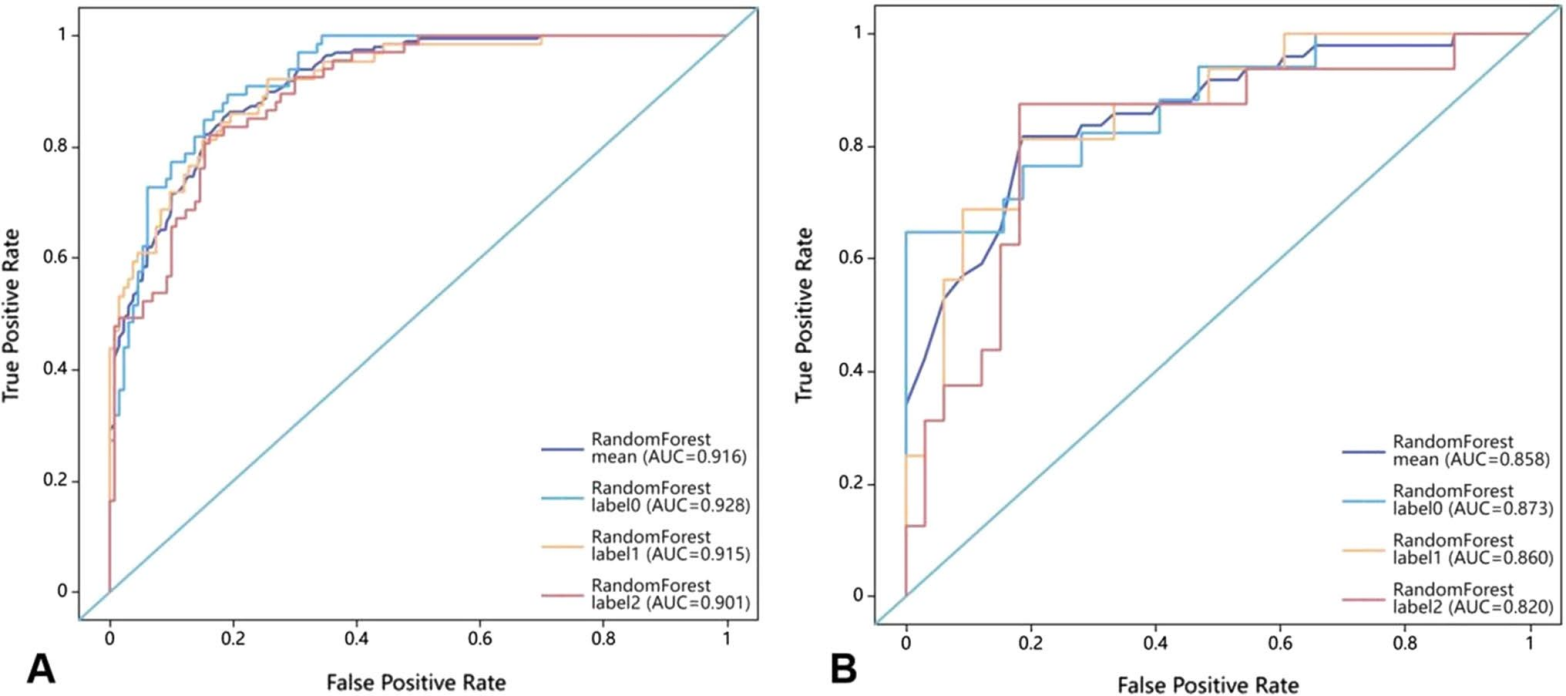
Receiver operating characteristic curves of the three-class classification radiomics model in the training set (**A**) and testing set (**B**). Labels 0, 1, and 2 represent low-MKI, intermediate-MKI, and high-MKI, respectively

**Fig. 8:**
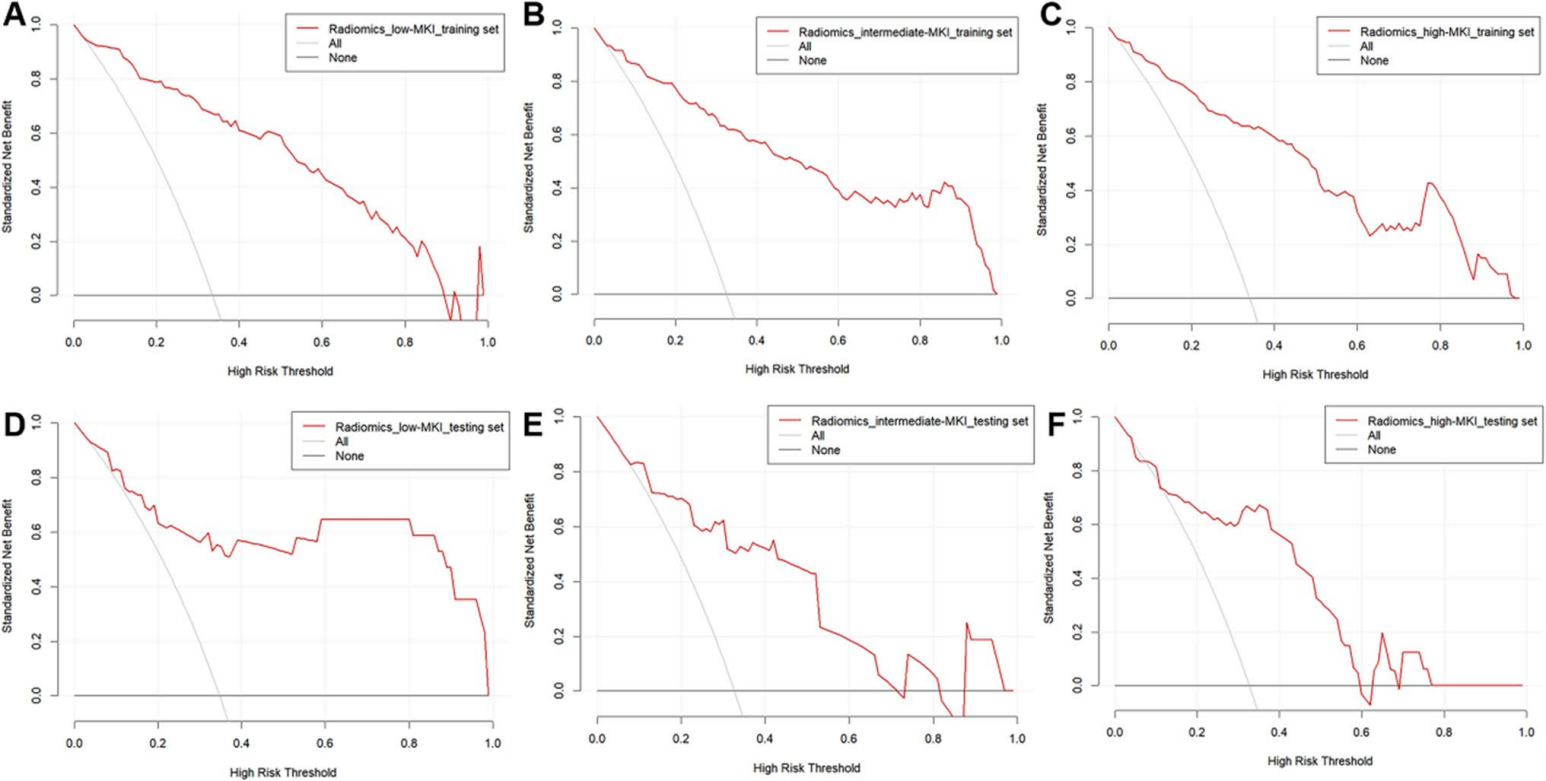
The clinical decision curves of the radiomics model for predicting the mitosis karyorrhexis index in the training and testing sets. **A**–**C** indicate the decision curves of the radiomics model for predicting the low-MKI, intermediate-MKI, and high-MKI, respectively, in the training set; **D**–**F** indicate the decision curves of the radiomics model for predicting the low-MKI, intermediate-MKI, and high-MKI, respectively, in the testing set

The mean AUC of the radiomics model was 0.916 (95% CI 0.913–0.921) in the training set and 0.858 (95% CI 0.841–0.864) in the testing set. Specifically, the radiomics model achieved AUCs of 0.928 (95% CI 0.927–0.934), 0.915 (95% CI 0.912–0.919), and 0.901 (95% CI 0.900–0.909) for predicting low-MKI, intermediate-MKI, and high-MKI, respectively, in the training set. In the testing set, the radiomics model achieved AUCs of 0.873 (95% CI 0.859–0.882), 0.860 (95% CI 0.852–0.872), and 0.820 (95% CI 0.813–0.839) for predicting low-MKI, intermediate-MKI, and high-MKI, respectively. Table 3 shows the detailed evaluation indexes of the radiomics model in the training and testing sets. The ROC curves of the radiomics model in the training and testing sets are illustrated in Fig. 7. Figure 8 illustrates the DCA curves of the radiomics model in the training and testing sets, indicating the clinical utility of the constructed radiomics model.

**Table 3:** Diagnostic performance of the three-class classification radiomics model

	AUC_training	AUC_testing	Sensitivity_training	Sensitivity_testing	Specificity_training	Specificity_testing	Accuracy_training	Accuracy_testing
Mean	0.916 (0.913–0.921)	0.858 (0.841–0.864)	0.873 (0.867–0.878)	0.844 (0.835–0.853)	0.742 (0.730–0.753)	0.674 (0.653–0.695)	0.829 (0.824–0.835)	0.788 (0.777–0.798)
Low-MKI	0.928 (0.927–0.934)	0.873 (0.859–0.882)	0.903 (0.898–0.908)	1.000 (1.000–1.000)	0.707 (0.695–0.718)	0.644 (0.621–0.666)	0.836 (0.830–0.842)	0.876 (0.868–0.885)
Intermediate-MKI	0.915 (0.912–0.919)	0.860 (0.852–0.872)	0.895 (0.890–0.900)	0.916 (0.906–0.926)	0.697 (0.685–0.708)	0.503 (0.479–0.526)	0.831 (0.826–0.836)	0.780 (0.769–0.791)
High-MKI	0.901 (0.900–0.909)	0.820 (0.813–0.839)	0.820 (0.813–0.827)	0.616 (0.600–0.632)	0.822 (0.811–0.832)	0.876 (0.860–0.892)	0.820 (0.814–0.826)	0.706 (0.694–0.718)

The numbers in brackets represent 95% confidence intervals

## Discussion

MKI is a critical component of the INPC system and an independent prognostic factor for neuroblastoma. Accurate assessment of MKI status in neuroblastoma is important for making informed treatment decisions. However, the current preoperative method for determining MKI status, which involves tumor puncture biopsy, has limitations due to its invasiveness, susceptibility to sampling errors resulting from necrotic tissue, spatial and temporal heterogeneity within the tumor, and subjectivity of manual counting. Previous studies have demonstrated these limitations [9, 30]. In the past few years, radiomics has emerged as a useful and objective technique to identify associations between radiomics features and tumor biomarkers, offering valuable insights into the pathological and clinical characteristics of lesions without the need for invasive procedures [31, 32].

To ensure reliable and repeatable radiomics features, two pediatric radiologists manually delineated the ROI in this study. The ICC analysis was conducted on the delineated ROI, and only features with an ICC greater than 0.80 were retained. In the study, 5 independent features of the intra-tumoral ROI were identified, with a significant contribution from texture features in the radiomics model. Texture features were derived from analyzing the relative spatial distribution and intensity levels of voxels to characterize the structural texture of two-dimensional or three-dimensional images [33]. Numerous studies have demonstrated that texture features provide valuable insights into tumor differentiation, pathological typing, radiogenomics, prognostic prediction, and more, as they reflect structural texture differences in intratumoral tissue anatomy [34–36].

Our study revealed that the most frequently selected radiomics features were texture features. These texture features were found to differ among the low-MKI, intermediate MKI, and high-MKI groups, indicating variations in gray uniformity and heterogeneity of the CECT images among lesions with different MKI statuses. This discrepancy may be attributed to the rapid proliferation of tumor cells at high-MKI status, resulting in uneven density and texture within the tumor on CECT images. The rapidly proliferating cells may also induce changes in tumor tissues. Additionally, tumors in specific areas may exhibit varying degrees of enhancement due to alterations in vascular structures during proliferation.

In conjunction with radiomics analysis, we examined clinical characteristics and determined that there was no noteworthy association between the age at diagnosis, gender, calcification, midline crossing, urinary VMA, and MKI status. These findings align with those of Feng et al. [22]. To predict high-, intermediate-, and low-MKI statuses of intra-tumoral regions of interest, the random forest machine learning method was utilized in this study. The mean AUC values for the radiomics model in the training set and the testing set were 0.916 (95% CI 0.913–0.921) and 0.858 (95% CI 0.841–0.864), respectively. These results demonstrate the robustness and predictive capability of our model in differentiating MKI statuses within the tumor regions of interest.

There are some limitations to this study that should be noted. First, as it was a single-center study, future multi-center studies are necessary to enhance the generalization and effectiveness of the radiomics model. Second, the CECT examinations of the included cases were conducted by two different manufacturers, which may impact the effectiveness of the model, despite our preprocessing of the images before extracting radiomics features. Third, the question of whether MRI could provide additional information worth further investigation remains. Last, we only compared two commonly imaging features among the MKI status in this study, i.e. calcification and across midline. However, the results showed that these two imaging features differed no significantly among low MKI, intermediate MKI, and high MKI groups. It is acknowledged that the comprehensive comparison of conventional features was not achieved in this study, because this study focused on the radiomics analysis.

## Conclusion

In conclusion, this study demonstrates the potential of utilizing radiomics features extracted from intra-tumoral CECT images to predict the MKI status in neuroblastoma. Texture features are shown to be more effective in distinguishing between lesions with different MKI statuses. These findings suggest that CECT radiomics features are correlated with MKI status and are helpful in reflecting the proliferation status of neuroblastoma cells. However, to validate these findings and address the limitations of this study, multi-center studies and further research with larger sample sizes are needed.

### Supplementary Information

Supplementary Material 1.

Supplementary Material 2.

## Data Availability

The datasets generated or analyzed during the study are available from the corresponding author on reasonable request.
