# Supplementary material for: The relationship between contrast-enhanced computed tomography radiomics features and mitosis karyorrhexis index in neuroblastoma
Source: Discov Oncol. 2024 Jun 1;15:201. doi: 10.1007/s12672-024-01067-0 (PMC11144178; doi:10.1007/s12672-024-01067-0)
Supplement: Supplementary file 2 — Supplementary Material 2. [file 12672_2024_1067_MOESM2_ESM.docx]

| **Criteria** | **Points** |
| --- | --- |
| 1.Image protocol quality - well-documented image protocols (for example, contrast, slice thickness, energy, etc.) and/or usage of public  image protocols allow reproducibility/replicability | +1 |
| 2.Multiple segmentations - possible actions are: segmentation by different physicians/algorithms/software, perturbing segmentations by (random) noise, segmentation at different breathing cycles. Analyse feature robustness to segmentation variabilities | +1 |
| 3.Phantom study on all scanners-detect inter-scanner differences and vendor-dependent features. Analyse feature robustness to these sources of variability | +1 |
| 4.Imaging at multiple time points - collect images of individuals at additional time points. Analyse feature robustness to temporal variabilities (for example, organ movement, organ expansion/shrinkage) | 0 |
| 5.Feature reduction or adjustment for multiple testing - decreases the risk of overfitting. Overfitting is inevitable if the number of features exceeds the number of samples. Consider feature robustness when selecting features | +3 |
| 6.Multivariable analysis with non radiomics features (for example, EGFR mutation) - is expected to provide a more holistic model. Permits correlating/inferencing between radiomics and non radiomics features | +1 |
| 7.Detect and discuss biological correlates- demonstration of phenotypic differences (possibly associated with underlying gene–protein expression patterns) deepens understanding of radiomics and biology | +1 |
| 8.Cut-off analyses - determine risk groups by either the median, a previously published cut-off or report a continuous risk variable. Reduces the risk of reporting overly optimistic results | 0 |
| 9 .Discrimination statistics - report discrimination statistics (for example, C-statistic, ROC curve, AUC) and their statistical significance (for example, p-values, confidence intervals). One can also apply resampling method (for example, bootstrapping, cross-validation) | +1 |
| 10.Calibration statistics - report calibration statistics (for example, Calibration-in-the-large/slope, calibration plots) and their statistical significance (for example, *P*-values, confidence intervals). One can also apply resampling method (for example, bootstrapping, cross-validation) | +1 |
| 11.Prospective study registered in a trial database - provides the highest level of evidence supporting the clinical validity and usefulness of the radiomics biomarker | 0 |
| 12.Validation - the validation is performed without retraining and without adaptation of the cut-off value, provides crucial information with regard to credible clinical performance | +2 |
| 13.Comparison to ‘gold standard’-assess the extent to which the model agrees with/is superior to the current ‘gold standard’ method (for  example, TNM-staging for survival prediction). This comparison shows the added value of radiomics | +2 |
| 14.Potential clinical utility - report on the current and potential application of the model in a clinical setting (for example, decision curve analysis). | +2 |
| 15.Cost-effectiveness analysis - report on the cost-effectiveness of the clinical application (for example, QALYs generated) | 0 |
| 16.Open science and data - make code and data publicly available. Open science facilitates knowledge transfer and reproducibility of the study | 0 |
| Total points (36=100%) | 16 (16/36, 44%) |
